# The design and implementation of a longitudinal social medicine curriculum at the University of Vermont’s Larner College of Medicine

**DOI:** 10.1186/s12909-021-02533-x

**Published:** 2021-02-24

**Authors:** Raghav K. Goyal, Christina A. Dawson, Samuel B. Epstein, Richard J. Brach, Sheridan M. Finnie, Karen M. Lounsbury, Timothy Lahey, Shaden T. Eldakar-Hein

**Affiliations:** University of Vermont’s Larner College of Medicine, UVMMC, 111 Colchester Ave, Smith 2, Burlington, VT 05401 USA

**Keywords:** Social determinants of health, Medical education, Undergraduate medical education, Preclinical medical education, Clinical medical education, Health care, Racism, Health inequities

## Abstract

**Background:**

Despite an abundant literature advocating that social determinants of health (SDH) be taught during undergraduate medical education, there are few detailed descriptions of how to design and implement longitudinal core curricula that is delivered to all students and accomplishes this goal.

**Methods:**

In this paper, we describe the design and implementation of a social medicine curriculum at the University of Vermont’s Larner College of Medicine (UVM Larner). Using Kern’s principles, we designed a longitudinal curriculum that extends through both preclinical and clinical training for all students and focused on integrating SDH material directly into basic science and clinical training.

**Results:**

We successfully developed and implemented two primary tools, a “Social Medicine Theme of the Week” (SMTW) in preclinical training, and SDH rounds in the clinical setting to deliver SDH content to all learners at UVM Larner.

**Conclusions:**

Extensive student-faculty partnerships, robust needs assessment, and focusing on longitudinal and integrated SDH content delivery to all students were key features that contributed to successful design and implementation.

**Supplementary Information:**

The online version contains supplementary material available at 10.1186/s12909-021-02533-x.

## Background

Social determinants of health (SDH) influence innumerable patient care outcomes from health care expenditures to life expectancy and professional satisfaction [1–5]. Physician educators are motivated to implement SDH as a central feature of undergraduate medical education [6–14]. This training has the potential to prepare future providers to notice the SDH, to gain tools to address them, and to reduce cognitive dissonance and “betrayals of purpose” [15] whereby SDH may exacerbate burnout through clinical productivity-driven incentives, helplessness about chronic health problems, and lack of insurance coverage [2, 16]. Despite growing awareness of the tolls of healthcare disparities in the United States [17], significant obstacles to meaningful inclusion of SDH training in the undergraduate medical curriculum remain. These obstacles include the surfeit of foundational science material considered necessary for medical training, diverse faculty opinions about the educational utility of teaching SDH topics, and stricter average evidentiary standards for incorporation of SDH material into the curriculum compared to what justified the inclusion of traditional foundational science material [18–20].

Building on prior publications describing creative means of incorporating SDH into undergraduate medical education [14, 21–24], and linked to relevant curriculum objectives required by the Liaison Committee on Medicine Education (LCME) such as “Societal Problems” and “Cultural Competence and Health Care Disparities” [25], we describe the design and implementation of a novel longitudinal core curriculum in social medicine at the University of Vermont’s Larner College of Medicine (UVM Larner).

This social medicine curriculum (SMC) includes diverse topics related to SDH and health disparities which utilize critical reflection, the incorporation of community voices, and experiential learning as essential teaching modalities. The UVM Larner SMC is deployed longitudinally to all learners, integrated directly into both didactic and clinical training, positions students as active learners who critique the existing health care system, engages them in interprofessional education, and is robustly assessed [21, 23, 26–29].

## Steps in the design of a novel social medicine curriculum

### Problem identification and needs assessment

In 2018, a group of student advocates at UVM Larner, including the Class of 2021 student authors, were concerned that their medical education was not adequately preparing them to recognize or redress the SDH. These concerns drove the group to advocate for change. This group adopted the name Social Justice Coalition (SJC) and set out to use Kern’s six steps of curricular development to advocate for change at UVM Larner [30]. Kern’s process for curricular development has been used with success in various domains of medical education [31–33] and offers a logical and stepwise methodology for curricular development that spans problem identification and needs assessment to implementation and evaluation.

Students initially identified the problem through firsthand experience of both preclinical and clinical curricula, which constituted their first, informal phase of needs assessment. Thereafter, students undertook a second, formal, lecture-by-lecture needs assessment which involved an analysis of slides and teaching materials.

These needs assessments led students to identify key stakeholders (Table 1). Patients are the primary stakeholders in that all efforts to teach the SDH in medical training are geared at addressing the health burdens faced by patients at large: disproportionate health effects on Black and Brown peoples, suicidality in transgender populations, mounting health care costs and poor insurance access, the chronic health issues created by mental illness, poverty, and poor access to resources for illness prevention, and the structural forces which cause and exacerbate substance use.

**Table 1:** Initial needs assessment and summary of stakeholders

Stakeholder	Needs assessment findings
**Patients**	- Central stakeholder in medical education
	- Impacted by racism, homophobia, sexism, etc. in the clinic
	- Impacted by rising healthcare costs and poor access to healthcare
	- Impacted by poverty and access to preventive health resources
	- Impacted by lack of cultural perspective, and humanism/connection in the clinic
**Students**	- Understanding SDH might mitigate burnout long-term
	- No preconceived notions about how healthcare delivery takes place
	- Likely more versed in SDH concepts than faculty
	- Many passionate students willing to build/develop curricula
	- Heavy workload, already stressed about board exams and content load
**Faculty/Course Directors**	- Make decisions with regards to education content and scheduling
	- Beholden to LCME standards and administrative expectations
	- Limited time to develop curricula due to academic and clinic responsibilities
	- Many passionate faculty who are excited to partner with students
	- Some resistant faculty who do not see how or why to change classical teaching
	- Faculty can serve as learners to educate themselves about this material
**Administration**	- Responsibility to ensure curricula is universal, regularly assessed, and adhering to LCME standards
	- Many competing priorities for curricular time
	- A number of passionate administrators who are willing to allow students to take lead with curricular development
	- UVM Larner has created environment that is willing to adjust and change

Further identification of faculty and administrators as joint stakeholders led to a targeted needs assessment phase. Key faculty, students, and course directors were invited to a series of meetings aimed at answering three key questions: Do you believe SDH is being adequately taught in the classroom? Where are the SDH currently being taught in the classroom? Where do you think there could be greater improvement of SDH education in the classroom?

**Fig. 1:**
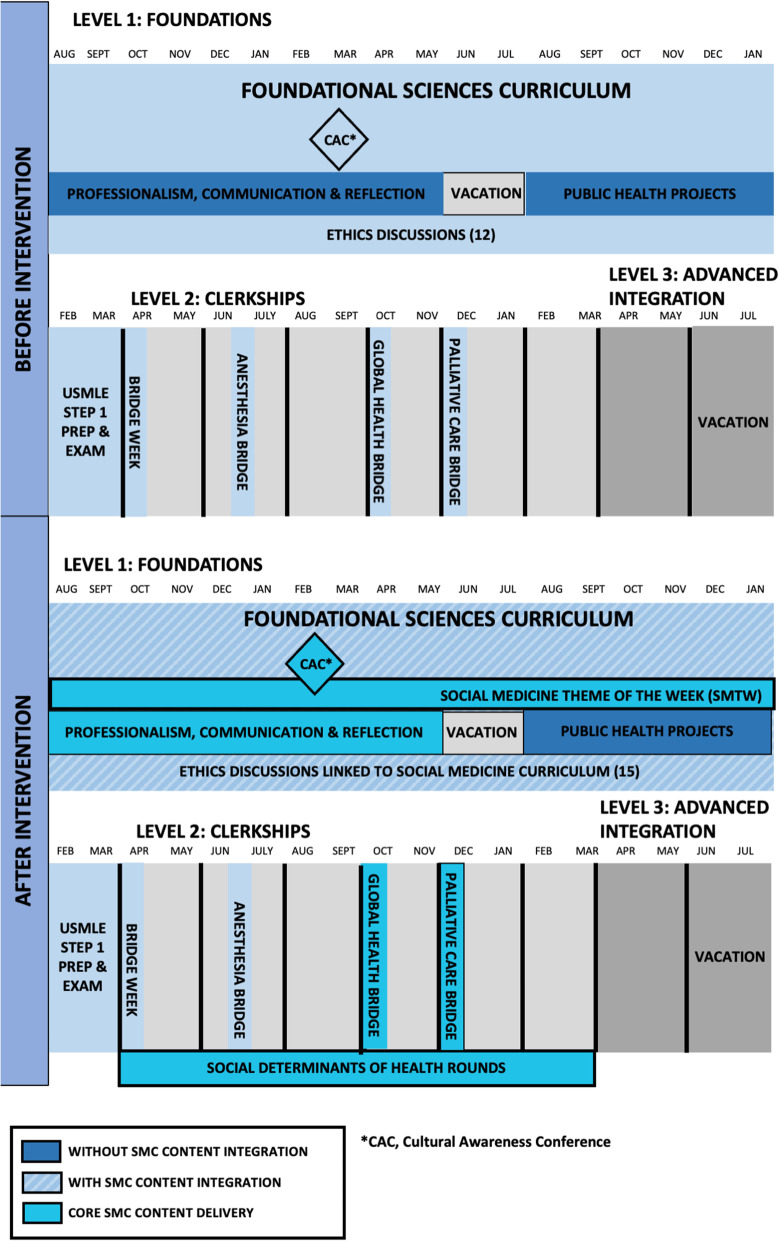
Vermont Integrated Curriculum Pre and Post Social Medicine Intervention

This needs assessment – which we estimate took approximately 10 h per week for several months – yielded two results: the formalization of a team of core students and faculty committed to this work and a clear map of where SDH content was already being taught (Fig. 1).

The content map allowed us to see what was being taught and to identify gaps. There were many SDH topics that UVM Larner was already teaching: an orientation seminar about racism and bias, a first-year weekly small group discussion course called Professionalism, Communication, and Reflection (PCR), two public health lectures in the first 2 years of training, a 5 month Public Health Project with a community organization during the second year, 12 ethics lectures in the first 2 years of training, two clinical “bridge weeks” on Palliative Care and Global Health, and an annual interprofessional 4-h Cultural Awareness Conference.

The extensive needs assessment and mapping process cemented the student and faculty team and revealed key opportunities for further intervention. For example, although SDH content existed at UVM Larner, this content was separated from the foundational sciences curriculum in discrete courses, informally assessed, lacked space for critical reflection, had no centralized coordination, and lacked direct clinical integration.

### Goals and objectives of a social medicine curriculum

Encouraged by how much material was already being taught, the students and faculty developed a new SDH curriculum with the goal of training a cohort of critically reflective medical students who would be prepared to see and address the complex social manifestations of health burden today. We named this improved curriculum the Social Medicine Curriculum (SMC). This term alludes to the societal and public health perspectives that traditionally fall under the banner of SDH but also emphasizes the importance of critical consciousness and humanities training as articulated in the Social Medicine Consortium’s consensus statement [34].

The design of the SMC started with the development of seven broad SMC learning objectives categories (Table 2) as well as 184 related session learning objectives (Supplemental 1). These objectives were generated through student and faculty conversations and literature review.

**Table 2:** Social medicine curriculum learning objectives categories

1. Investigate your own health and wellness through a Social Medicine lens.	
2. Appraise the history of American healthcare and health insurance systems.	
3. Support the importance of the Social Determinants of Health and understanding a patient’s social context when providing care.	
4. Appraise the intersection of Social Determinants of Health with the history, perspective, and experience of specific marginalized populations.	
5. Defend the importance of cultural differences in health and how they affect health care outcomes	
6. Synthesize the role of the United States in the Global Health narrative.	
7. Assemble available tools and strategies to advocate for social change	

These learning objectives focus on encouraging critical self-reflection and giving students the historical and narrative tools necessary to contextualize health disparities. The overarching goal is to improve patient outcomes by training a cohort of future physicians that is prepared to enter into a healthcare landscape that is increasingly governed by financial, political, and social forces, and to equip them with the interpersonal and narrative tools necessary to navigate this landscape to understand each patient’s unique challenges in achieving their best health outcomes.

Critical self-reflection is an important tool for physicians embarking on work to redress the SDH. Being able to navigate and appraise one’s own place in SDH issues such as systemic racism, housing insecurity, and homophobia for example is prerequisite to understanding material that does not have clear right or wrong answers, advocating for patients, and mitigating burnout [13, 14]. Much of our work rested on the presence of the weekly small group discussions held already in the PCR course as space for SDH-focused critical reflection.

Upon completion of the needs assessment and learning objectives, we set out to design and implement a social medicine curriculum that was critically reflective, featured community voices, was longitudinally deployed to learners, integrated directly into existing preclinical and clinical curriculum, co-championed by diverse faculty, and regularly assessed. This curricular design involved three core organizing processes: Cross-Curricular Integration, Content Generation, and Clinical Social Determinants of Health Rounds.

### Cross-curricular integration of preclinical training

To highlight its central importance to undergraduate medical education, and to ensure it would be given equal curricular footing with other foundational sciences, we focused on cross-curricular integration of the SMC. This involved two main processes: (1) drawing connections between existing SM curricular elements and the foundational sciences and (2) an intervention we call the Social Medicine Theme of the Week (SMTW). For each week of the first and second years of the preclinical curriculum, we developed a Social Medicine Theme, grounded in one of our seven broad SMC learning objectives categories (Table 2), and directly connected to what was being taught in the foundational science curriculum.

The creation of the SMTW allowed us to not only to draw topical connections between the foundational sciences and the SMC but also to link foundational science courses to the PCR course. This in turn allowed small groups of students to discuss and analyze SMC topics in more detail. PCR is a small group discussion course that meets for approximately 90 min weekly for the duration of the pre-clinical curriculum. Moderated by a faculty member, the course covers a broad range of topics from the role of medical interpreters, to abortion, to burnout and racism. The SMC harnessed the potential of PCR as a multidisciplinary discussion space where complicated topics could be reflectively discussed in a brave [35] and non-judgmental space.

The cross-curricular integration of SMC with foundational sciences and PCR is depicted by examples shown in Table 3. Beyond creating topical linkages between foundational sciences, PCR and the SMC, students volunteered to announce the SMTW each week to their class. This moment of student-led reflection, which typically lasts less than 5 min, draws student attention to curricular framing, and, as appropriate, to the SMC’s goal of creating future physicians who are leaders of health care change. The SMTW announcements positioned students as active leaders of the curriculum integration process. We included a reminder on students’ online calendars, asked course directors to notify faculty of the SMTW, and facilitated the addition of new aligned slides or discussion questions into key sessions. This material was linked to exam questions, case studies in active learning sessions, and the SJC tied the SMTW to extracurricular events such as lunch discussions around complementary topics.

**Table 3:** Summary of cross-curricular integration of social medicine theme of the week with professionalism, communication and reflection course, foundational sciences topics and ethics

Relevant foundational science curriculum topics	Social Medicine Theme of the Week	Social Medicine Curriculum topic addressed in Professionalism, Communication and Reflection course	Ethics topics
Sickle cell anemia, population genetics, basics of genetics	The genetic basis of race	Eugenics and the culture of medicine	Introduction to ethics including foundational bioethics values like justice
Non-mendelian genetics, gross anatomy lab	Death and dying	Cadaver, death, and dying	
Cancer, white coat ceremony, genetics	Power and privilege	Power, privilege, and the white coat	History of ethical violations in medicine including via physician disrespect for vulnerable populations
Distribution of “doctor bags” Annual named lecture on equity (topic: Transgender health) PCR spiritual care shadowing orientation	The power of listening	Narrative Medicine and visit to hospital	Solidarity with vulnerable populations
Cystic fibrosis, respiratory anatomy and physiology, sympathetic chain	History of American healthcare	Introduction to American healthcare	Respect for patient autonomy: medical decision-making, surrogate decision-making and shared decision-making
GI anatomy and physiology, gross anatomy lab	Race and food	Racism and food	Ethical approaches to race and culture
Reproductive system anatomy and physiology	Owning bodies	Abortion and values clarification	Abortion, physician assisted death, euthanasia and approaching disagreements about topics in medicine
Toxicology, lead poisoning	Housing and water	Case studies in environmental health (Flint, Michigan, Dakota Access, climate change)	Decision support for wise resource allocation
Anticoagulants	The economics of healthcare	Doctors and money, pharmaceutical pricing, and cost to patients	Health care system ethics, wise allocation of transplanted organs
Virology, HIV	The AIDS crisis	History of the AIDS epidemic	
Microbiome, chronic diarrhea, micronutrients, parasites	Domestic access to food and water	American food policy, mapping of Burlington, Vermont, food deserts	
Food as medicine, lipids	Stigma and sexuality	Sexuality and medicine (w/ body image and medicine)	Sexual health ethics
Brain tumors	The mythology of the physician	Burnout & self-Care (+ cultural perceptions of doctors, internal perceptions of doctors, competition, workaholism, etc.)	Wellbeing and ethics
History of opioids	Addiction, stigma, recovery	Doctors, substance abuse, & recovery	
Parkinson’s, tremor, Huntington’s	Aging and bias	Ageism and social isolation	

This curricular scaffolding created opportunities for course directors to incorporate SM content into existing sessions. Table 4 depicts one example of how the SMTW created opportunities to integrate SM content directly into 1 week of basic sciences curriculum.

**Table 4:** Snapshot of Social Medicine Theme of the Week (SMTW): housing and water

Classroom sessions	Activity Type	Social Medicine Curriculum Intervention
PCR: “Case Studies in Environmental Health”	Small group Session	Creation of new PCR session. See List 2.
Orientation session	Lecture	Course director introduced the SMTW and summarized its relevance
Introduction to Clinical Decision Making	Small group Session	Addition of new lecture slides acknowledging social determinants of health in clinical decision making, addition of new group discussion questions.
Hematopoiesis	Lecture	*None*
Heme, Globin, and RBC Maintenance	Case-based Learning	*None*
Introduction to Anemia	Case-based Learning	*None*
Anemia of Decreased Production	Case-based Learning	*None*
Anemia of Decreased Red Cell Survival	Case-based Learning	*None*
Toxicology	Case-based Learning	Case Study: Flint Michigan, addition of new prework material, objectives, and readiness quiz questions
Introduction to Hemostasis	Lecture	*None*
Hemostasis	Team-Based Learning	*None*
Anemia	Small group Session	Addition of discussion questions addressing environmental exposures and social determinants of health resulting in anemia
Week 1 Formative Quiz	Formative Quiz	Addition of multiple-choice questions addressing Flint case study, social causes/consequences of anemia

During this week, we established a SMTW titled “Housing and Water” based on the basic science content related to toxicology and microcytic anemias. The cross-curricular integration was realized first by the development of a new PCR session called “Environmental Health: Flint and Dakota Access” (Table 5) Then, a lecture slide was inserted into the Toxicology session about how poverty, nationality, and race make certain populations more prone to environmental harm with two associated learning objectives. Further, we posed slides into the “Clinical Decision Making” lecture that asked students to reflect on their assumptions about patient’s access to housing and basic human rights based on their physical appearance in the clinical setting. This week, we also wrote two multiple choice exam questions that would appear on students’ exams.

**Table 5:** PCR session on case studies in environmental health

Relevant Basic Sciences Material: Introduction to toxicology, microcytic anemia	
**PCR Learning Objectives**	
1. Explore the role of our healthcare system in mediating environmental health issues.	
2. Explore the responsibilities a physician has in addressing health concerns with environmental underpinnings.	
3. Examine which populations of people are more vulnerable to environmental exposures.	
**PCR Readings/Resources**	
1. “Everything Water Touches,” (Video, https://vimeo.com/158843832)	
2. “Flint’s Fight for American Children,” (Video, https://www.youtube.com/watch?v=pJQvNbYeSws)	
3. “DAPL and American Indian as ‘Protector’,” (Article, https://medium.com/hindsights/https-medium-com-hindsights-in-hindsight-american-indian-as-protector-8f11a325dab7).	
**PCR Discussion Questions**	
1. What are the Structural/Social Determinants of Health that might lead to lead poisoning?	
2. What are some long-term social impacts that lead poisoning can have on communities and families?	
3. What is the role of a physician in responding to acute lead poisoning?	
4. Who is most vulnerable to environmental exposures, both nationally and internationally?	

We performed an identical process as described above for each of the first- and second-year courses (first year seen in Supplement 2), with the goal of drawing connections between SM content and the basic sciences curriculum, assuring the content had a place to be discussed meaningfully and reflectively.

### Preclinical content generation

The above work of cross-curricular integration allowed us to link many SDH-related topics to existing foundational science topics. Some content of the ideal SMC drafted through our needs assessment and learning objective generation was not yet covered in the existing UVM Larner curriculum, thus requiring new content generation.

One example of Content Generation was already discussed in the prior section, specifically the creation of a novel PCR session titled “Environmental Health: Flint and Dakota Access” (Table 5). Another example was during a SMTW titled “The Genetic Basis of Race.” The theme was based on the week’s basic science training in genetics and served to highlight eugenics and the University’s historical participation in this movement. The PCR session was entitled “Eugenics and the Culture of Medicine” and can be seen in Table 6.

**Table 6:** P CR session titled “Eugenics and the culture of medicine”

Relevant basic science material: genetics	
**PCR Learning Objectives**	
1. Discuss how what is condoned and promoted within the culture of medicine is in a constant state of social reevaluation.	
2. Understand the history of eugenics at the University of Vermont and in the state of Vermont.	
3. Understand the genetics of race and how it has been used to justify mistreatment.	
4. Examine communication in the context of medicine.	
**PCR Readings/Resources**	
1. Description of eugenics in Vermont: http://www.uvm.edu/~lkaelber/eugenics/VT/VT.html	
2. Passage from “Breeding better Vermonters” by Nancy Gallagher	
3. Donald Berwick’s article, “The epitaph of profession”: https://www.ncbi.nlm.nih.gov/pmc/articles/PMC2629825/	
4. Description of the Eugenics Survey in Vermont: http://www.uvm.edu/~eugenics/famstudiesf.html	
**PCR Sample Discussion Questions**	
1. What is the definition of race? Is race genetically acquired? How has science/genetics been used to justify mistreatment?	
2. Who are the Abenaki people?	
3. How has medicine and the practice of medicine changed over time?	
4. Where does science end and social perception begin?	
5. How is the history of eugenics at Vermont a useful way to think about how cultural expectations of the role of medicine are constantly changing?	
a. Are there things that are currently happening in medicine today that might seem problematic with the benefit of hindsight?	
6. History of medicine and doctors is one of silencing different types of people and instituting certain types of people, white men in suits, as physicians and silencing others	
7. Do you know of any other examples of the institution of medicine causing more harm than good?	
8. Is forced sterilization ever justified?	
9. How is the inequitable distribution of resources in the healthcare system similar to the eugenics movement?	
10. How do modern attempts to interfere with genetics resemble or not resemble eugenics movements of the past?	

When generating new content, we used diverse resources from the medical humanities such as literature, visual arts, music, podcasts, policy, and law. Throughout we featured voices from outside of medicine and paired these resources to discussion questions that fostered critical self-reflection as a means of enhancing professionalism and clinical competency as well as challenging the expectation that complex questions need to have simple answers [13, 28, 36].

During the design of the SMC, we believed it was critically important to recruit community members and other healthcare professionals into educator positions [21]. Prior to our intervention, PCR already featured a number of sessions that featured diverse voices including nurses, physicians dealing with substance use, refugee interpreters, transgender community members, and others. Such opportunities to transform community members with complex lived experience into educators is an essential element of humanizing SM content, revealing how issues like poverty, substance abuse, racism, and homophobia truly impact health [37].

New content generation ultimately led to the creation and/or revision of 17 PCR sessions as well as the revision of two Public Health lectures, a substantive redesign of a dozen ethics sessions in the Foundations of Clinical Sciences course, the creation of dozens of small group discussion questions for the foundational science first-year courses, and hosting a variety of extracurricular lunch-time sessions on current events.

### A clinical social medicine curriculum: preliminary stages

Clinical integration of SDH training is an essential feature of any undergraduate medical curriculum [21]. The clinical environment offers students the opportunity to see the reality of how SDH principles impact patient lives.

Based on our initial needs assessment we noticed the same issues with SDH training in the clinical environment: the content was sparse, separated from the rest of the curriculum, and informally assessed.

To address these shortcomings, we performed a literature review to look for tools that could be used to deliver SDH training in the clinical environment. Interventions included those that did not directly relate to caseload in clinical environment [36, 38, 39] such as teaching kitchen approach [20], which demonstrated that experiential cooking and nutrition education improved trainees’ abilities to counsel patients on diet and nutrition. In addition, the review revealed a number of approaches that focused on a select cohorts of students [21, 22, 40–42]. Ultimately, we turned to a Longitudinal Integrated Curriculum which focused on using a rounds based technique [43] that required students actively reflect on how the SDH impact their patient caseload, and to create space for students to discuss these issues with peers and faculty.

With this framework in hand, students and faculty met with clerkship directors to elaborate the intervention to their particular contexts. SDH rounds are currently in an early implementation phase in the Psychiatry and OB/GYN clerkships. Discussion with other clerkship directors is currently ongoing.

## Discussion

In response to national mandates to prepare physicians to resist racism and other forms of injustice in medicine, and following a local institutional needs assessment informed by national literature of social medicine, we created a social medicine curriculum at UVM Larner by drafting 184 SMC learning objectives housed in seven overarching categories, devising the SMTW as an effective platform for cross-curricular integration, generating new curricular content, and using SDH rounds to integrate this content into the clinical environment.

This work aimed to train students to recognize and redress the SDH in a fashion that utilized existing resources, focused on teamwork, and could offer a practical process that could be generalizable to other institutions. Key lessons learned during the creation of the UVM Larner SMC include the pivotal importance of small group critical reflection on SMC content, the strategic importance of extensive faculty engagement, the value of longitudinal curricular integration extending into clinical training, and initial reflections on SMC assessment [21, 24].

When revising or creating new sessions for the SMC, we focused on opportunities for students to develop skills in critical reflection. Critical reflection is essential to SDH training in that it encourages students to centralize their own role in health inequity, reflect on burnout, and to understand that forces like poverty, racism, patriarchy, and climate change are human-made forces without clear solutions [14].

The already existing Professionalism, Communication, and Reflection (PCR) course at UVM Larner created space for critical reflection and served as the foundation for integration into the basic science curriculum. Our initial needs assessment and onboarding of faculty and administrators identified the potential of PCR in our work, and established allies who would allow us to expand and grow it. Institutions that do not have a comparable longitudinal reflection course might struggle to generalize our work to their institution, but the needs assessment and faculty engagement that led us to PCR could yield alternative opportunities.

Faculty support and involvement as stakeholders has been the central reason for our success. Course directors, administrators, and faculty leadership have provided invaluable feedback and mentorship through every phase of design and implementation, they performed tasks that would have been impossible for students, including speaking directly with lecturers, posting SMTW announcements weekly in the students’ online calendars, and keeping students apprised of logistical concerns. After the initial cohort of students has graduated, faculty members can ensure the curriculum continues, and that lessons in implementation are not forgotten between class years. Our collaborations reinforced our belief that much of this work needs to be driven by faculty and staff with content expertise, institutional knowledge and longevity, and tangible support for the ongoing work of curricular leadership.

Though our work began with students at UVM Larner, we believe this process could begin at other institutions with faculty. Our needs assessment and early exploration phase cemented our student and faculty team, and this process could have similar successes elsewhere.

To further support faculty, we have hosted a half-day social medicine teaching session, and one grand rounds on social medicine education. Moving forward, we plan to establish regular expert-led faculty training in facilitation skills and SDH content to ensure that small group discussion sessions and SDH rounds allow for reflective and nonjudgmental exploration. This form of facilitation is not a formal part of faculty training and is essential to a healthy and growing SDH curriculum. Incorporating faculty development as an integral part of the continued improvement of this curriculum will also help establish sustainability as students’ involvement is transient by nature.

We attended closely to the importance of assessment in the design of the SMC, frequently assisting course directors in creation of multiple-choice exam questions that linked to SMC material. That said, reflective essays and observed performance in clinical settings may be superior to multiple choice questions in assessing understanding of social medicine material and impact on patient outcomes [22, 44, 45]. Our nascent SDH clinical rounds offer faculty the opportunity to explore topics with students, and to require students to make formal SDH presentations which, in the future, can be evaluated by objective standards.

Longitudinal curricular integration is a central strength of the SMTW approach to social medicine teaching. The SMTW has allowed us to pinpoint where social medicine content fits naturally into the basic science curriculum and creates efficient means to develop discussion questions, slides, and exam questions. This strategy makes social medicine feel like an integral part of medical training. Despite our integration success so far, there are still many weeks of the curriculum where the SMTW is not sufficiently integrated outside of the PCR session for that week.

The SMTW approach has also made it possible to deploy our curriculum universally to all students. The literature cites a number of models featuring SM tracks for select students [21, 22, 40–42], and although these models offer the potential of deeper training for students who are destined to become leaders in healthcare, we believe they miss an opportunity to build capacity and awareness in all future physicians.

### Next steps

There are two forms of evaluation that are needed at this time: a programmatic evaluation of our work thus far), and a longitudinal evaluation to assess the impact of the curriculum on students’ future practice as physicians. Does talking about racism in a small group session result in students recognizing their own role in various systems of oppression? Does it equip students with the tools to make change? Do SDH rounds give students tools to improve patient care, affect change, and reduce burnout?

Despite successes in fostering critical self-reflection and involving voices from the community in PCR, we have yet to incorporate service-learning into the curriculum, which is an essential task for any social medicine curriculum. Soon, we hope to unlock cross-curricular integration of an existing community-based course called the Public Health Project to fully harness the experiential power of service learning in the SMC [24].

In the clinical environment, we have so far succeeded in integrating SDH rounds into two out of eight core clerkships. We hope to continue to work with clerkship directors to explore SDH rounds as a modality for teaching Social Medicine, or to find other clinical tools that will allow students to apply a Social Medicine lens to the patients for which they care.

As essential next steps in improving our educational methods, we plan to continue to build our SMTW through preclinical training, offer expert-led faculty training, expand our clinical SDH rounds, and begin assessment of impacts on burnout, student reflections, and patient outcomes. We recognize that the clinical years offer unique challenges due to constantly changing schedules, geographic and temporal diversity, and demanding hours of training, but we believe that the use of small group sessions similar to the PCR groups featured earlier in the curriculum have potential to facilitate ongoing reflection upon clinical experiences by third- and fourth-year students.

## Conclusion

We identify several major contributors to the successful design of the novel SMC at UVM Larner, including a student-led needs assessment, the Social Medicine Theme of the Week, the utilization of a year-long small group discussion space for critical reflection, the creation of SMC learning objectives, commitment to cross-curricular integration with foundational sciences, and adjunctive events such as SDH rounds. These processes can be generalized to other institutions with different baseline foundational science curricula and SDH-related content, and we are dedicated to ensuring durable student-faculty collaborations that empower future physicians to be effective agents of change and critical advocacy in health care. This work is not done. Next steps include curricular evaluation and faculty development. As Audre Lorde once said, “revolution is not a one-time event” [46].

## Supplementary Information

**Additional file 1: Supplemental 1.** Full 184 learning objectives of the Social Medicine Curriculum at Larner College of Medicine. **Supplemental 2.** Summary of all first year cross-curricular integration of SMTWs with PCR and Foundational Sciences.

## Data Availability

The datasets used and/or analysed during the current study are available from the corresponding author on reasonable request.

## Abbreviations

LCME: Liaison Committee on Medical Education
PCR: Professionalism, Communication, and Reflection course
SDH: Social Determinants of Health
SJC: Social Justice Coalition at Larner College of Medicine
SMC: Social Medicine Curriculum
SMTW: Social Medicine Theme of the Week
URM: Underrepresented in Medicine
UVM Larner: University of Vermont Larner College of Medicine
