# Correction to: The design and implementation of a longitudinal social medicine curriculum at the University of Vermont’s Larner College of Medicine

**DOI:** 10.1186/s12909-021-02594-y

**Published:** 2021-03-10

**Authors:** Raghav K. Goyal, Christina A. Dawson, Samuel B. Epstein, Richard J. Brach, Sheridan M. Finnie, Karen M. Lounsbury, Timothy Lahey, Shaden T. Eldakar-Hein

**Affiliations:** University of Vermont’s Larner College of Medicine, UVMMC, 111 Colchester, Ave, Smith 2, Burlington, VT 05401 USA

**Correction to: BMC Med Educ 21, 131 (2021)**

**https://doi.org/10.1186/s12909-021-02533-x**

Following publication of the original article [1], the authors informed us that Fig. 1 has been labeled as “Before intervention” and “Before intervention” instead of “Before intervention” and “After intervention”.

The original article has been corrected.

## References

[1] Goyal (2021). The design and implementation of a longitudinal social medicine curriculum at the University of Vermont’s Larner College of Medicine. BMC Med Educ.

