# Supplementary material for: The design and implementation of a longitudinal social medicine curriculum at the University of Vermont’s Larner College of Medicine
Source: BMC Med Educ. 2021 Feb 24;21:131. doi: 10.1186/s12909-021-02533-x (PMC7903930; doi:10.1186/s12909-021-02533-x)
Supplement: Supplementary file 1 — Additional file 1: Supplemental 1. Full 184 learning objectives of the Social Medicine Curriculum at Larner College of Medicine. Supplemental 2. Summary of all first year cross-curricular integration of SMTWs with PCR and Foundational Sciences. [file 12909_2021_2533_MOESM1_ESM.docx]

**The Design and Implementation of a Longitudinal Social Medicine Curriculum at the**

**University of Vermont’s Larner College of Medicine**

Raghav K. Goyal*, Christina A. Dawson*, Samuel B. Epstein*, Richard J. Brach,

Sheridan M. Finnie, MPH, Karen M. Lounsbury Ph.D, Timothy P. Lahey MD, MMSc, Shaden T. Eldakar-Hein MD, MS

From the University of Vermont’s Larner College of Medicine, Burlington,

VT 05405

*Authors contributed equally

Correspondence should be addressed to:

Tim Lahey, MD, MMSc

Professor of Medicine & Director of Ethics

University of Vermont Larner College of Medicine

111 Colchester Ave, Smith 2

Burlington, VT 05401

Phone: (802) 847-4594

E-mail: [Timothy.Lahey@med.UVM.edu](mailto:Timothy.Lahey@med.UVM.edu)

**Supplemental 1: Full 184 learning objectives of the Social Medicine Curriculum at Larner College of Medicine:** [**Full 184 learning objectives of the Social Medicine Curriculum at Larner College of Medicine**](https://docs.google.com/document/d/1NI2yEgW4WxokM2j3Oxn-WVYBY6jnx_o6uGCdeRBshic/edit?usp=sharing)

1. **Investigate your own health and wellness through a Social Medicine lens.**
   1. Discuss the privilege and power inherent in becoming a physician.
   2. Demonstrate a willingness to discourse critically about your own background and the backgrounds of others in the medical profession.
   3. Demonstrate a willingness to discourse about personal bias.
      1. Explicitly name Racism, Sexism, and Homophobia.
      2. Fat bias
      3. Attractiveness bias
      4. Age bias
      5. Illness bias
      6. Geriatric bias
   4. Describe the culture of medicine.
      1. Identify how the culture of medical education contributes to the persistence of stereotypes and bias in medicine.
      2. Understand how the culture of medicine contributes to clinical decision making.
         1. Identify how representations of normal v. pathological may influence patient care.
      3. Identify that the demographics of physicians are not the same as the general patient population and the social and clinical ramifications of this.
         1. Identify the challenges that physicians may face in understanding the struggles of their patients.
         2. Identify the demographic breakdown of physicians and physician leadership in America today and understand which populations are under-represented.
      4. Recognize the history of “pimping” and not treating residents and trainees well.
      5. Describe the factors that contribute to physician burnout.
         1. Understand RVUs and the obstacles that physicians face in spending adequate time with patients.
         2. Describe the landscape of EMRs and computer documentation that adds to the stress of practicing medicine.
         3. Describe the sense of hopelessness that physicians experience in trying to tackle population-level health burdens in the healthcare setting.
      6. Discuss the elevated status of physicians in the United States and in local communities, which empowers them as advocates and conveys responsibility on each of them individually.
      7. Discuss how what is condoned and promoted within the culture of medicine is in a constant state of social reevaluation.
   5. Discuss humanism in medicine as a concept and the specific ways to practice as a humanistic physician.
2. **Appraise the history of American healthcare and health insurance systems.**
   1. Compare and contrast the U.S. healthcare system with that of other countries.
      1. United Kingdom, France, Germany, Canada, Taiwan, Switzerland, Cuba, Japan, South India, and others
   2. Summarize a brief history of Medicare and Medicaid and the differences in each program.
      1. Understand that American healthcare has a long and complex history that is constantly changing.
   3. Summarize the history of major health care reforms and reform attempts in the United States, including the ACA.
      1. Beginnings of health insurance
      2. Passage of Medicare and Medicaid
      3. Affordable Care Act
      4. Current events/issues
   4. Summarize the healthcare reform efforts in Vermont, both historical and current.
   5. Understand American healthcare focus on curative over preventative healthcare interventions and the long-term consequences on health
   6. Critique the role that pharmaceutical companies play in the American Healthcare system.
      1. Describe the history and practice of pharmaceutical advertising.
      2. Explain the history of opioids as it relates to pharmaceutical companies.
      3. Analyze how pharmaceutical companies determine the cost of drugs.
      4. Critique pharmaceutical companies’ drive to garner high profits.
   7. Analyze the history of biomedical research ethics.
      1. Understand the history of eugenics at the University of Vermont and in the state of Vermont.
      2. Analyze the story of Henrietta Lacks and her mistreatment by researchers.
      3. Describe seminal case examples in biomedical research ethics violations, e.g. Tuskegee Syphilis Study.
      4. Analyze the populations represented and not represented in biomedical research through the lens of the Framingham study.
      5. Contrast discrepancies in funding for diseases affecting different populations, e.g. sickle cell disease v. cystic fibrosis; erectile dysfunction v. endometriosis.
   8. Explore how medicalization results in cost inflations that impacts patients, medical staff, social and medical institutions.
   9. Explore how the creation of the American Healthcare System displaced other forms of local and generational forms of healing.
3. **Support the importance of the Social Determinants of Health and understanding a patient’s social context when providing care.**
   1. Examine the connection between economic stability and health.
      1. Explore how health, work, family, transportation, housing, and nutrition are intimately linked when living in poverty.
      2. Contrast different cultural experiences of poverty faced by different populations.
   2. Examine the connection between education and health.
      1. Understand the importance of early sexual education and the challenges of having children early in life.
      2. Understand the challenges faced by patients with different reading levels.
      3. Define and contrast “literacy” and “health literacy.”
   3. Examine the connection between community context and health.
      1. Explore the different ways that discrimination can affect one’s health.
      2. Understand the importance of understanding social supports for a patient, and develop sensitivity to different models of social support.
      3. Assess the role that Adverse Childhood Experiences and trauma play in development and future health outcomes.
      4. Understand how mass incarceration has changed lives of certain populations.
      5. Examine the importance of strong social cohesion on health and what stands in the way of building social cohesion in different neighborhoods.
   4. Examine obstacles that stand in the way of healthcare access.
      1. Define what community resources can be described as “health resources.”
      2. Understand how health insurance, or lack thereof, restricts access to healthcare.
      3. Examine how health resources are distributed in communities and who has access to them.
      4. Understand the importance of transportation when thinking about appointments and access, with attention to how this impacts people with disabilities.
      5. Examine how immigration status can be an obstacle in accessing healthcare.
      6. Examine how being a non-English speaker can compromise a patient’s ability to access healthcare.
   5. Examine the connection between housing, the built environment, and health.
      1. Understand that homelessness is a complicated status that different people enter into for different reasons.
      2. Summarize the history of red-lining as a tool to disenfranchise certain populations, and how redlining still operates today.
      3. Define and discuss the complexity of gentrification and its effects on housing access.
      4. Understand how age and maintenance of a home, including its paint, plumbing, and water source, contribute to the risk of exposure to lead and resulting lead toxicity.
      5. Understand how the built environment, such as where street lights, sidewalks, parks, and playgrounds are located, contributes to health.
      6. Understand how the built environment impacts physical activity and the subsequent impact on health.
      7. Give examples of how water rights have been compromised for vulnerable populations domestically and around the world, and identify responsible parties.
   6. Examine the obstacles faced by various populations in accessing nutritious food.
      1. Define “food desert.”
      2. Explain why food habits established early in life are hard to change and use personal examples.
      3. Understand some historical reasons that food high in caloric content and low in nutritional value is cheap.
      4. Summarize the history and efficacy of sugar taxes as a public health policy around the world.
      5. Define the “Hunger Vital Sign” and its use as a screening tool.
4. **Appraise the intersection of Social Determinants of Health with the history, perspective, and experience of specific marginalized populations.**
   1. Define Health Disparities and the Social Determinants of Health.
   2. Examine the lasting and intergenerational impact that slavery has had on institutions, communities, and individual health.
      1. Understand the impact that the stress of racism has on communities.
      2. Distinguish between “implicit bias,” “racism,” and “systemic racism,” and understand how all three play a role in America today.
      3. Understand the genetics of race and how it has been used to justify mistreatment.
      4. Understand how epigenetics leads to intergenerational transmission of illness.
      5. Understand how African American pain is often underestimated or ignored by medical professionals.
   3. Examine the lasting and intergenerational impact of colonialism on the health of indigenous communities in the U.S.
      1. Demonstrate a brief understanding of the historical struggles faced by Native populations in the Americas.
         1. Describe the diaspora and history of dispossession of the Abenaki peoples who were displaced by settlers in Vermont.
      2. Understand the history and location of Reservations and how access to resources on them is limited.
      3. Understand how limited access to food resources on reservations contributes to health problems in Native communities.
      4. Describe the factors that contribute to alcoholism and illicit drug use in Native Communities.
      5. Outline the environmental disasters that have disproportionately affected Native Populations in America today.
   4. Examine how mass incarceration and racist incarceration affect communities and individual health.
      1. Summarize a brief history of the War on Drugs and the expansion of minority incarceration in the prison system.
      2. Discuss the role of police brutality in regards to communities’ trust of law enforcement, feeling of safety, and mental and emotional health.
      3. Articulate how incarceration can disrupt family and community structure and stability.
      4. Identify the ethics, social, legal, and healthcare issues that affect prison populations.
      5. Describe the healthcare system in prisons and jails and the barriers to providing care.
   5. Examine the health inequities faced by members of the LGBTQ+ community.
      1. Understand the social and medical context in which members of the LGBTQ+ community seek healthcare.
      2. Understand the history of the AIDS Crisis and its reflection of Federal neglect of the LGBTQ+ community.
      3. Understand the difference between sex, gender identity, and gender role.
      4. Appreciate the emotional and physical challenges to living Openly today.
      5. Understand the options available today for individuals in Transition.
      6. Explain why older and younger members of the LGBTQ+ community may differ in their experience of their identity.
      7. Understand how the medical community’s perception of LGBTQ+ status has changed over time.
      8. Summarize strategies to promote culturally competent care for LGBTQ+ patients.
   6. Examine the health inequities faced by women due to their specific needs around sexual and reproductive health and how lack of adequate resources to afford care disproportionately impacts women.
      1. Understand that all factors of gender inequity--including limited access to education, legal systems that fail to protect women, gender-based violence, and the wage gap--are exacerbated by poverty and are often logistical barriers to accessing health care.
      2. Understand how gender norms impact health burdens women face.
         1. Understand how HIV, cardiovascular disease, depression, and anxiety disorders differentially affect women versus men.
         2. Understand how female pain is often underestimated or disregarded by medical professionals.
      3. Explain how legal, political, and cultural concerns over women’s reproductive health can influence or negatively impact access to accurate and adequate information and access to health care.
         1. Understand the systematic bias in our healthcare system that had made reproduction and childbirth inherently more dangerous as a woman of color.
         2. Discuss the dissonance between the medical concept of bodily autonomy and the current barriers to access reproductive healthcare.
         3. Clarify and inquire about your individual views, perceptions, and biases about abortion and reproductive healthcare.
      4. Examine how sexism is experienced differently by different minority groups.
   7. Examine the obstacles that individuals living in rural settings face in achieving equitable healthcare.
      1. Identify the prohibition of using federal dollars to conduct research on gun violence.
   8. Discuss the significance of cultural and historical differences in dietary norms and preferences along with their implications on long-term health.
   9. Examine historical trends in the treatment and care of the mentally ill and what challenges lie ahead.
      1. Demonstrate familiarity with Dorothea Dix and her advocacy work for the mentally ill, and the shortcomings of early advocacy work.
      2. Identify the intersection between mental illness and homelessness.
      3. Discuss the changing definitions and perceptions of mental illness, and the challenges of viewing the brain as just another organ.
      4. Understand that Substance Use Disorder is a disease and not a lapse of personal character, and understand the challenges faced by care providers in treating it.
   10. Examine how environmental hazards have disproportionately affected certain vulnerable populations in America, and around the world today.
       1. Explain the factors that led to the Standing Rock protests.
       2. Examine how response efforts to Hurricane Katrina were not equitably distributed.
       3. Define “environmental racism” in the context of the Flint water crisis.
       4. Discuss how extreme weather events negatively impact community access to healthcare in the context of the disproportionate allocation of resources between US territories (Puerto Rico, US Virgin Islands, Guam) and the continental US.
   11. Examine the unique healthcare needs of elderly populations and the barriers they experience in achieving quality care.
       1. Discuss the importance of compassionate end-of-life care.
   12. Examine the unique health concerns that come up in ensuring equitable healthcare for children and adolescents.
5. **Defend the importance of cultural differences in health and how they affect health care outcomes.**
   1. Explore the discipline of Narrative Medicine.
      1. Appreciate the physician’s unique role in sharing in others’ suffering.
   2. Understand that health, wellness, sickness, and death are culturally defined phenomena.
      1. Explore Traditional Chinese Medicine, acupuncture, herbal medicine, etc. as examples of healthcare that have adequately provided for patients long before, and in conjunction with, allopathic medicine.
      2. Understand the importance of understanding a patient’s cultural background when making diet and lifestyle recommendations.
   3. Explore patient examples where the doctor’s definition of health and the patient’s definition of health may be different and how that influences interventions.
      1. Recognize that what may be seen as a disease in one health perspective may be a gift in another (deafness, psychiatric “disorders” etc.).
      2. Recognize how religion can change perspectives and expectations about healthcare.
      3. Discuss how stigma affects perceptions of illness and influences individual’s decisions to seek--or not to seek--care.
   4. Explore the expectations of doctors to “fix” everything, give pills, shots etc., to preserve life indefinitely.
   5. Recognize the role that palliative care plays in helping to achieve patients’ goals.
   6. Describe how stories and narrative medicine can be used to build trust and understanding between different groups.
6. **Synthesize the role of the United States in the Global Health narrative.**
   1. Understand that the economic value of an individual’s life changes based on their nation of origin and the conditions of their birth.
      1. Understand how research funding for different diseases depends on which countries and peoples are most affected by an illness.
   2. Explore how Western Colonialism has affected and continues to affect global healthcare systems and health outcomes.
      1. Explore American interventionism including but not limited to the following countries: Haiti, Guatemala, Argentina, Vietnam, Laos, Cambodia, the Philippines, Iraq, Afghanistan, the Marshall Islands, Nigeria, West Papua, Liberia, the Congo etc.
         1. Examine America’s use of Agent Orange in Vietnam and the long-term effects of its use on health.
         2. Understand the effects of American nuclear testing on the health and well-being of those living in the Marshall Islands.
      2. Define and summarize the meaning of “neoliberal capitalism.”
      3. Explore the United States economic and military involvement in the continued occupation of Palestine, and the tools used by the USA to make the struggle intractable.
   3. Discuss the role of Climate Change with respect to its effects on health outcomes.
      1. Define the “anthropocene” and understand the effects of human activity on the climate.
      2. Explore how the health of human beings is intimately tied to the health of other living organisms and ecosystems.
      3. Understand how America’s actions contribute to Climate Change.
      4. Describe how certain populations are disproportionately affected by climate change.
   4. Explore the short and long-term implications of medical humanitarianism.
      1. Contrast the effects on populations receiving aid with the effects on populations providing aid.
   5. Examine the role of education in developing sustainable systems of self-improvement.
   6. Understand the historic role of the U.S. in influencing the health and well-being of individuals in other countries.
   7. Identify differences between roles of governmental health organizations and NGOs in providing aid.
   8. Explore cultural discrepancies and limitations in trying to promote Western Medicine across a global network.
   9. Understand the importance of including the affected community in the development of community health interventions.
   10. Discuss the responsibility of nations to provide aid during times of conflict.
       1. Discuss this responsibility with respect to conflict resulting from international intervention, versus internal instability.
   11. Evaluate organ transplant sourcing and availability with respect to patient income, nationality, and social class.
   12. Understand how research funding for different diseases depends on which countries are most affected by an illness.
7. **Assemble available tools and strategies to advocate for social change.**
   1. Understand the importance of experiential learning in gaining fluency in Social Medicine.
   2. Dialogue about the role of a physician in addressing population-level struggles and disparities.
   3. Understand the clinical importance of and learn how to use social screening tools like the Hunger Vital Sign and ACEs tool.
   4. Understand the role of a physician as a part of a multidisciplinary team consisting of healthcare workers, social workers, and community organizations to support patients during and after their time in the healthcare setting.
   5. Understand the breadth and depth of community resources available to Vermonters, including where such resources may be lacking, and practice utilizing said resources.
   6. Understand and practice using Motivational Interviewing as a tool to encourage patients to take control of their own health outcomes.
   7. Understand the role of unions and the impact that they have on healthcare practitioners.
   8. Explain how the research paradigm of Community Based Participatory Research (CBPR) can be used to perform research in vulnerable communities.
   9. Explore different forms of activism and different ways that physicians can play a role in shaping social, cultural, and global institutions.
   10. Explore how organizations advocating for issues related to racism, housing, food security, and other social goals, are complementary to the practice of medicine.
   11. Learn how to engage in the local, state, and federal legislative processes.

**Supplemental 2: Summary of all first year cross-curricular integration of SMTWs with PCR and Foundational Sciences**

| **Relevant foundational science curriculum topic** | **Social Medicine Theme of the Week** | **Social Medicine Curriculum topic addressed in Professionalism, Communication and Reflection course** |
| --- | --- | --- |
| Orientation | None | Introduction to PCR |
| Sickle cell anemia, Introduction to ethics, population genetics, Basics of genetics | The Genetic Basis of Race | Eugenics and the Culture of Medicine (formerly Culture of Medicine and Communication) |
| Introduction to public health, HIV screening, medical ethics | The Social Determinants of HIV | Group Decision Making: HIV |
| Non-mendelian genetics, gross anatomy lab | Death and Dying | Cadaver, Death, and Dying |
| Gross anatomy lab, Integrated metabolism, Muscular dystrophy | NONE | Mentor Meetings |
| Gross anatomy lab, Muscular dystrophy | Narrative Medicine | A Family Story and Family Centered Care |
| Genomics, medical ethics, Cancer, pharmacogenomics, cytogenetics, cell cycle, DNA repair | Rural Health | *SJC Lunch Talk: Rural Health* |
| Cancer, White Coat, Genetics | Power and Privilege | Power, Privilege, and the White Coat (formerly Professionalism and the White Coat) |
| Distribution of “doctor bags” Annual Imbasciani lecture (topic: Transgender health) PCR spiritual care shadowing orientation | The Power of Listening | Narrative Medicine and Visit to Hospital |
|  | NONE | NONE |
| Immune system basics, cardiovascular anatomy and physiology | Health Disparities and Cardiovascular Disease | *SJC Lunch Talk + Effective Teams* |
| Cystic Fibrosis, respiratory anatomy and physiology, sympathetic chain | History of American Healthcare | Introduction to American Healthcare |
| Renal anatomy and physiology, | "But race is the child of racism, not the father." | None (Mandatory Ta-Nehisi Coates talk) |
| GI anatomy and physiology, gross anatomy lab | Race and Food | Racism and Food |
| Reproductive system anatomy and physiology | Owning Bodies | Abortion and Values Clarification |
| Endocrine anatomy and physiology, skull anatomy | Stress and Identity | Diversity, Sexuality, and the LGBTQ Community |
| Autonomics, cranial nerves, gross anatomy lab | Burnout | Physicians, Medical Students, and Wellness |
| Pharmacokinetics | None | None |
| Toxicology, lead poisoning | Housing and Water | Case Studies in Environmental Health (Flint, Michigan, Dakota Access, Climate Change) |
| Anticoagulants | The Economics of Healthcare | Doctors and Money (Pharmaceutical Pricing) // Cost to Patients |
| Bacteriology | Vulnerable Populations and Infectious Disease | *SJC Lunch Talk + Mentor Meetings* |
| Vaccinology, mycobacteria | Screening and Global Healthcare Access | Medical Interpreters & Refugee Health (New American Health Screening) |
| Virology, HIV | The AIDS Crisis | History of AIDS Epidemic |
| Neoplasia | None | None |
| Cultural Awareness Conference (topic: ) | [Tie to Cultural Awareness Workshop] | Doctors and Nurses (some tie in with Cultural Awareness Conference) |
| Infectious diarrhea, micronutrients | Access to Food and Water (global) | Food and Health |
| Microbiome, chronic diarrhea, micronutrients, parasites | Access to Food and Water (domestic) | American Food Policy, Mapping of Burlington, food deserts |
| None | None | Careers in Medicine |
| Hepatitis, cirrhosis, helminths | Social Determinants of Hepatitis | Substance Abuse and Stigma |
| Lecture: Food as Medicine, lipids | Stigma and Sexuality | Sexuality and Medicine (w/ Body Image and Medicine) |
| Diabetes | Social Determinants of Diabetes | Case Studies in Diabetes: African Americans and Pima Indians |
| Diabetes continued | None | None |
| None | None | Mentor Meetings |
| Brain tumors | The Mythology of the Physician | Burnout & Self-Care (+ cultural perceptions of doctors, internal perceptions of doctors, competition, workaholism, etc.) |
| Seizures, CNS infections, cranial nerves | Cultural Perception of Disease | *SJC Lunch Talk + Public Health Projects* |
| History of Opioids lecture | Addiction, Stigma, Recovery | Doctors, Substance Abuse, & Recovery |
| Parkinson's, tremor, Huntington’s | Aging and Bias | Ageism and Social Isolation |
|  | NONE | Closure |
| Auditory system | Defining Disability | *SJC Lunch Talk: Deaf culture* |
| Schizophrenia, anxiety, mood | History of Mental Health | *SJC Lunch Talk: History of US Mental Health resources* |
| Consciousness, limbic disorders | Limits of Modern Medicine | *SJC Lunch Talk: God, Science, Consciousness* |
